# Impulsivity and compulsivity as parallel mediators of emotion dysregulation in eating‐related addictive‐like behaviors, alcohol use, and compulsive exercise

**DOI:** 10.1002/brb3.2458

**Published:** 2021-12-20

**Authors:** Emma Forsén Mantilla, David Clinton, Elin Monell, Johanna Levallius, Andreas Birgegård

**Affiliations:** ^1^ Department of Medical Epidemiology and Biostatistics Karolinska institutet Stockholm Sweden

**Keywords:** addictive‐like behaviors, compulsivity, emotion dysregulation, impulsivity, mediation

## Abstract

**Introduction:**

Transdiagnostically relevant psychological traits associated with psychiatric disorders are increasingly being researched, notably in substance use and addictive behaviors. We investigated whether emotion dysregulation mediated by impulsivity and/or compulsivity could explain variance in binge eating, food addiction, self‐starvation, and compulsive exercise, as well as alcohol use (addictive‐like behaviors relevant to the obesity and eating disorder fields).

**Method:**

A general population sample of adults (*N *= 500, mean age = 32.5 years), females (*n *= 376) and males (*n *= 124), completed the Difficulties in Emotion Regulation Scale‐16, the Trait Rash Impulsivity Scale, the Obsessive‐Compulsive Inventory—Revised, the Eating Disorders Examination Questionnaire, the Self‐Starvation Scale, the Exercise Dependence Scale, the Yale Food Addiction Scale, and the Alcohol Use Disorders Identification Test online. Besides gender comparisons and intercorrelations between measures, we used predefined multiple mediation models with emotion dysregulation as independent variable, impulsivity and compulsivity as parallel mediators, to investigate whether these factors contributed explanatory power to each addictive‐like behavior as outcome, also using age and body mass index as covariates.

**Results:**

Females scored higher than males on emotion dysregulation and the eating‐related addictive‐like behaviors food addiction, self‐starvation, and binge eating. Intercorrelations between measures showed that emotion dysregulation and compulsivity were associated with all outcome variables, impulsivity with all except compulsive exercise, and the eating‐related behaviors intercorrelated strongly. Mediation models showed full or partial mediation of emotion dysregulation for all behaviors, especially via compulsivity, suggesting a behavior‐specific pattern. Mediation models were not affected by age or gender.

**Discussion:**

Addictive‐like behaviors seemed to be maintained by trait levels of emotion dysregulation, albeit channeled via trait levels of compulsivity and/or impulsivity. The role of emotion dysregulation may help us to understand why addictive‐like behaviors can be difficult to change in both clinical and nonclinical groups, and may be informative for treatment‐planning in patients where these behaviors are present. Our findings support adopting a more dimensional approach to psychiatric classification by focusing psychological facets such as those studied.

## Graphical abstract

We studied behaviors affecting weight, relevant for, for example, the obesity and the eating disorders fields, to investigate whether emotion dysregulation mediated by impulsivity and/or compulsivity could explain variance in binge eating, food addiction, self‐starvation, and compulsive exercise, as well as alcohol use. Mediation models showed full or partial mediation of emotion dysregulation for all behaviors, especially via compulsivity, in a suggestive disorder‐specific pattern.

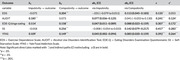

## 1 INTRODUCTION

### 1.1 The transdiagnostic approach and addictive‐like behaviors

The Research Domain Criteria (RDoC), developed by the US National Institute of Mental Health (NIMH), encourages researchers to go beyond traditional diagnostic classification systems and investigate new perspectives on psychiatric disorders integrating many levels of information (genetic, neurobiological, behavioral, psychological; https://www.nimh.nih.gov/research/research‐funded‐by‐nimh/rdoc/about‐rdoc). The idea is that mental health and illness may be better studied by exploring degrees of dysfunction in six major domains of functioning spanning the full range of human behavior, from normal to abnormal. Both symptoms of psychopathology, and the traits suggested to underlie them, predominantly vary along a continuum, and research on mental disorders has shifted focus from categorical to dimensional models (Brooks et al., 2012; Gillan & Seow, 2020; Markon et al., 2011; Wright et al., 2013). With regard to addiction and addiction‐like behaviors, several subconstructs in the RDoC could be used to conceptualize transdiagnostic processes implicated in such disorders (Brooks et al., 2017; Yucel et al., 2019). Such a transdiagnostic approach to the classification, etiology, and treatment of addictive and addictive‐like behaviors that emphasizes underlying psychological factors may be more theoretically and clinically relevant than prevailing diagnostic systems. Further, for example alcohol dependence, exercise dependence, gambling addiction, and binge eating show phenomenological, clinical, and neurobiological similarities (Kim & Hodgins, 2018), which raises the possibility that phenotypically distinct psychiatric symptoms are associated with similar psychological factors. Such findings are consistent with a transdiagnostic approach and also suggest that psychopathological phenomena of interest can fruitfully be studied in both clinical and community samples. In particular, impulsivity, compulsivity, and emotion dysregulation (all captured by various constructs in the RDoC; see e.g., Brooks et al., 2017) show promise for being associated with addictive and related behaviors (Abramowitz & Berenbaum, 2007; Pearson et al., 2015; Robbins et al., 2012; Schreiber et al., 2012; Wildes & Marcus, 2013). However, systematic research that examines all three constructs together in relation to such behaviors is lacking.

Weight‐affecting behaviors such as compulsive exercise, self‐starvation, food addiction, and binge eating have all been placed in the addictive‐like behaviors category (Adams, 2009; Davis, 2013; Eichen et al., 2016; Godier & Park, 2015). These behaviors have marked negative effects on psychological and physical health, and often result in unhealthy gain or loss of weight. Although positively associated in both clinical and community samples (Levallius et al., 2020; Lipson & Sonneville, 2017), and all within the domain of food intake/burn off and weight gain/loss, less is known about potential common functional attributes and psychological motivators. Emotion dysregulation has been suggested to be a trigger for these behaviors (Bunio et al., 2021; Haynos et al., 2017; Leehr et al., 2015; Meyer et al., 2011), but multiple functions and motivations may exist simultaneously. For example, compulsive exercise might be subjectively motivated by a wish to reduce weight and conform to socially accepted body ideals and avoid shame, while driven by compulsions to follow rigid, negatively reinforced routines.

### 1.2 Behavioral addiction and weight affecting addictive‐like behaviors

“Behavioral addictions,” or addictive‐like behaviors, denote problematic behaviors that share features of addictions as per criteria in the Diagnostic and Statistical Manual of Mental Disorders, 5th edition (DSM‐5) (American Psychiatric Association, 2013). These refer to behaviors engaged in compulsively while experiencing craving, or to avoid anxiety, negative affect, or withdrawal symptoms; they are associated with some degree of tolerance and are continued despite adverse physical, emotional, or interpersonal consequences. Similar to substance addictions, addictive‐like behaviors may also have initially been positively reinforced (i.e., inducing a “high” sense of accomplishment, positive emotion), but over time replaced by negative reinforcement, where not engaging in the behavior leads to distress, thus laying the basis for dependence. Although evidence suggests some associations between addictive‐like behaviors and psychiatric disorders (Starcevic, 2016), the etiological mechanisms of these behaviors and how they relate to one another are not clearly understood. In the present study, we investigated associations between psychological background factors and such behaviors in an attempt to inform clinical thinking and map out directions for future research.

### 1.3 Emotion dysregulation, impulsivity, and compulsivity

Emotion dysregulation, impulsivity, and compulsivity have been established as important transdiagnostic dimensions relevant for understanding both psychiatric disorders and addictive‐like behaviors (Mole et al., 2015; Pearson et al., 2015; Tiego et al., 2019; Yücel et al., 2019). Trait emotion dysregulation involves difficulties in emotional awareness, clarity, and acceptance, and in managing one's behavior and refraining from impulsive actions when in distress, and limited access to strategies to manage distressing emotions (Gratz & Roemer, 2004). Emotion dysregulation also describes momentary usage of strategies that for instance fail to sufficiently downregulate emotions or strategies with negative long‐term consequences (Aldao et al., 2010). The regulation of emotion has been proposed to be a central mechanism of addictive behaviors, such that engagement in addictive behaviors may function as a maladaptive strategy to temporarily regulate negative affect (including negative affect as a consequence of withdrawal), increasingly at the expense of more adaptive strategies to regulate emotions (Brooks et al., 2017). Impulsivity involves rash, poorly conceived actions with little thought to consequences that often result in negative repercussions, whereas compulsivity refers to maladaptive persistent behavior that is irrelevant to goals or the situation at hand (Robbins et al., 2012). Importantly, impulsivity and compulsivity may not be spectrum opposites, but distinct psychological factors that interact to shape specific behavioral expressions (Chamberlain et al., 2018; Robbins et al., 2012). They might be described as two ways of not being in control of thoughts, feelings and/or behaviors, thereby limiting the ability to engage in goal‐directed, planned action (Gillan, Kosinski, et al., 2016). Impulsive actions, particularly when in distress (i.e., “negative urgency”), may be of particular relevance for the initiation of maladaptive behaviors (Eben et al., 2020), including addictions and addictive‐like behaviors (Robbins et al., 2012). Further, over time these kinds of behaviors have been suggested to “hijack the reward system” via compulsivity, so that other, previously rewarding (or negatively reinforced via attenuation of negative affect) behaviors are replaced with the compulsive behavior (Brooks et al., 2017; Gillan, Kosinski, et al., 2016; Gillan, Robbins, et al., 2016). Compulsivity appears more likely to contribute to such entrenched behaviors in the presence of stress, distress and anxiety, potentially as these kinds of negative and assumingly dysregulated emotions, seem to increase the likelihood of overreliance on (maladaptive) habits (Gillan, Robbins, et al., 2016).

### 1.4 Maladaptive emotion regulation canalized via impulsivity and/or compulsivity

The centrality of emotion regulation may help explain why many specific addictive‐like behaviors are replaceable by other addictive‐like behaviors (Cooper et al., 1998). Consistent with this, a recent study found that different symptom behaviors may serve similar psychological functions and may therefore be substituted for each other during treatment (Garke et al., 2019). It has been suggested that there is likely to be specificity regarding which addictive‐like behaviors are more associated with impulsivity or compulsivity, respectively (Fineberg et al., 2014). A recent meta‐review suggested that while both constructs are implicated in a range of addictions (including behavioral), their role is not homogeneous across conditions (Lee et al., 2019). Further, one study suggested that exercise to regulate negative affect (i.e., similar to addictive behaviors) was related to a combination of poor trait emotion regulation and trait compulsivity in one female and one male college sample (Dreier et al., 2021). Thus, addictive‐like behavior may be explained by its emotion regulating properties as modified by trait levels of impulsivity and compulsivity. That is, the specific emotion regulating behaviors a person is likely to engage in may be affected by impulsivity and compulsivity, with the latter potentially promoting behaviors requiring perseverance and planning, such as compulsive exercise, and the former promoting engagement in behaviors that are triggered more by either internal emotional urgency or situational cues, such as drinking alcohol or overeating palatable foods (Dreier et al., 2021; Fineberg et al., 2014; Lee et al., 2019). Thus for example, a person with relatively higher impulsivity may be more likely to binge eat in the presence of unregulated anxiety, whereas a person with more compulsive tendencies may restrict eating trying to numb similar anxiety. Of clinical relevance, substitution may then be facilitated when the new behaviors that replace the old have similar impulsive and compulsive qualities, and thereby provide a good subjective “fit.” Therefore, it is of interest to understand which behaviors are related to which psychological factors, but the relationships between emotion dysregulation and these suggested traits (i.e., impulsivity and compulsivity) and how they in turn relate to weight affecting addictive‐like behaviors are not fully understood. Better knowledge of these relationships could benefit both the eating disorder‐ and the obesity fields.

### 1.5 Aim

In the present study, we tested a model where the experience of dysregulated emotion is hypothesized to create an urge to escape from or modify subjective discomfort by engaging in weight‐affecting addictive‐like behaviors, the likelihood of which varies by levels of trait impulsivity and compulsivity. We focus on the weight‐affecting behaviors mentioned above (i.e., self‐starvation, exercise dependence, food addiction, and binge eating), with the addition of problematic drinking in order to study potential differences between weight‐affecting addictive‐like behaviors and a more conventional form of addiction. Since a problem with previous empirical attempts to understand addictive‐like behaviors is that studies have largely focused exclusively on the impulsivity/compulsivity distinction without considering the role of emotion regulation, we aimed to test the mediating role of impulsivity and compulsivity in the association between emotion dysregulation as predictor and addictive‐like behaviors as outcome variables in a community sample.

## 2 METHOD

### 2.1 Participants and procedure

An online service listing opportunities for participation in research (Studentkaninen/Accindi; https://www.accindi.se/ [in Swedish]) was used to recruit 500 adult participants aged 18–71 (females *n *= 376; and males *n *= 124) who were mainly students (further descriptive data in Table 1). An automatic invitation email was sent by the service to 7774 registered and eligible participants (inclusion criteria: >18 years of age, living in the Stockholm area, understanding Swedish), with a link to online questionnaires created in Google Docs. The inclusion cap of 500 (based on power analysis for a separate study) was reached in 3 days; attrition is difficult to calculate since some may not have seen the email. Participants gave informed consent and reported age, gender, height/weight, and tobacco use (i.e., smoking and/or use of Swedish “snus”) before completing questionnaires, described below in the order they appeared. Three questionnaires were translated by the authors (noted below), with back‐translation by a native English speaker. Response time to the total of 132 items ranged from 15 to 20 min. Participants who supplied an email address or cell phone number (*N *= 460) received a 100 SEK gift certificate (approx. 12 USD). Ethical approval was given by the Stockholm Ethical Review Board (Dnr: 2016/2022‐31/2). The sample has been used in a previous study with different aims (Levallius et al., 2020).

**TABLE 1:** Mean (standard deviation), range, and gender comparisons (*t*‐tests, or Welch's *t* when variances were unequal) for all study variables. Cohen's *d* effect sizes are reported when *p* < .05

		Women		Men				
	Full sample	(*n *= 376)		(*n *= 124)				
Variable	*M* (*SD*)	*M* (*SD*)	Range	*M* (*SD*)	Range	*t* (men vs. women)	*p*	*D*
Age	32.5 (11.71)	32.6 (11.88)	18–69	32.0 (11.22)	19–71	0.49	.628	
BMI	24.3 (4.55)	24.1 (4.70)	15.9–45.0	24.7 (4.06)	16.6–37.9	−1.15	.252	
TRIS	1.2 (0.55)	1.2 (0.56)	0–2.8	1.2 (0.53)	0.11–2.8	1.22	.224	
OCI‐R	15.6 (10.95)	16.0 (10.76)	0–60	14.3 (11.46)	0–51	1.55	.123	
DERS‐16	5.0 (2.44)	2.6 (0.89)	1.0–5.0	2.1 (0.83)	1.0–4.3	5.20*	<.001	0.58
YFAS	0.6 (1.36)	0.8 (1.47)	0–7	0.3 (0.88)	0–7	4.28*	<.001	0.43
SS	10.1 (16.91)	11.4 (17.79)	0–96	6.0 (13.13)	0–80	3.68*	.001	0.35
AUDIT	5.6 (5.63)	5.6 (5.66)	0–34	5.8 (5.55)	0–23	–0.42	.675	
EDS	39.3 (19.20)	38.3 (18.63)	21–107	32.5 (20.60)	21–109	−2.14	.033	0.30
EDE‐Q Binge eating	2.8 (5.66)	3.4 (6.16)	0–30	1.2 (3.28)	0–20	5.07*	<.001	0.47

*Note*. For reference, OCI‐R clinical cutoff is 21 (Foa et al., 2002), AUDIT is 6 for women and 8 for men (Saunders et al., 1993), cutoffs have not been published for the DERS‐16, TRIS, SS, or EDE‐Q binge eating, and the YFAS and EDS have algorithm‐based cutoffs that do not simply rely on computed sums/means.

* Welch's *t* due to significant Levene's test.

BMI = body mass index; TRIS = Trait Rash Impulsiveness Scale; OCI‐R = Obsessive‐Compulsive Inventory—Revised; DERS‐16 = Difficulties in Emotion Regulation Scale‐16; YFAS = Yale Food Addiction Scale; SS = Self‐Starvation Scale; AUDIT = Alcohol Use Disorders Identification Test; EDS = Exercise Dependence Scale; EDE‐Q = Eating Disorders Examination Questionnaire.

### 2.2 Materials

*Difficulties in Emotion Regulation Questionnaire‐16* (DERS‐16; Bjureberg et al., 2016; Gratz & Roemer, 2004) measures emotion dysregulation in 16 items rated 1 (“almost never”) to 5 (“almost always”). Items form five subscales and one total score (sum of all items), with higher scores reflecting higher emotion dysregulation. The total score was used in analyses. The DERS‐16 has adequate psychometric properties (Bjureberg et al., 2016; Nordgren et al., 2020), and present total scale Cronbach's α was .95.

*Eating Disorder Examination Questionnaire* (EDE‐Q 6.0, 28 items; Fairburn & Beglin, 1994) measures cognitive ED symptoms during the past 28 days on a 0−6 scale, as well as categorical (yes/no) variables and frequencies of key symptoms (e.g., objective binge eating and self‐induced vomiting). The EDE‐Q has good psychometric properties, including concurrent validity (Berg et al., 2012). In the present study, Item 14 was used to measure binge eating (number of times during the past 28 days with episodes of binge eating) dichotomized into presence of binge eating (i.e., reponses of 0 vs. ≥1).

*Obsessive‐Compulsive Inventory—Revised* (OCI‐R; Foa et al., 2002) measures obsessive and compulsive tendencies in 18 items rated 0 = “not at all” to 4 = “extremely,” and items are summed to provide a total score. Cronbach's α in the current sample was .89.

*Trait‐Related Impulsivity Scale* (TRIS; Mayhew & Powell, 2014) is a 9‐item measure of trait impulsivity, rated 0–3 from “*rarely/almost never*” to “*almost always/always*.” Cronbach's α in the current sample was .80.

*Yale Food Addiction Scale* (YFAS, short‐version 9 items; Gearhardt et al., 2016) was translated for the study. YFAS measures addiction criteria based on DSM‐IV substance dependence in relation to food, scored 0 (never) to 4 (four or more times per week). Items include cravings, loss of control, withdrawal, continuation despite cessation attempts, and clinically significant impairment. YFAS can be computed as a symptom count of 0–7 symptoms, which was used here. Cronbach's α in the present sample was .89.

*Exercise Dependence Scale* (EDS, 21 items; Hausenblas & Downs, 2002) was translated for the study. EDS is based on the seven dependence criteria from DSM‐IV in relation to exercise. Items are rated 0 (never) to 6 (always), providing a mean score which was used in analyses. Cronbach's α in the present sample was .96.

*Self‐Starvation Scale* (SS, 16 items, translated for the project; Godier & Park, 2015) is based on the YFAS, but adapted to capture the compulsive element of self‐starvation, probing the same dependence criteria related to limiting food intake. Items are rated 0 (never) to 6 (every day) and the scale has good psychometric properties (Godier & Park, 2015). Cronbach's α for the mean total score in the present sample was .96.

*Alcohol Use Disorders Identification Test* (AUDIT; Saunders et al., 1993) comprises 10 items rated on a 5‐point scale and measures consumption of alcohol. Cut‐offs for unhealthy alcohol use were set at a score of 6 for males and 4 for females, as recommended by Johnson et al. (2013). The instrument is widely used and has good psychometric properties (Saunders et al., 1993). Cronbach´s α in our sample was .87.

### 2.3 Statistical analyses

Statistical analyses were performed in Statistical Package for the Social Sciences (SPSS) Statistics 26.0 for Mac. Scale correlations were computed using Pearson's *r*, while gender differences, performed to investigate whether to control for gender, were investigated using *t*‐tests, or Welch's *t* when Levene's test indicated unequal variances. In order to mitigate Type I error risk, we required *p *< .01 for statistical significance. Five mediation analyses, one for each outcome, with two parallel mediators (M_1_ and M_2_) were conducted to evaluate direct and indirect effects, using the PROCESS macro for SPSS by Hayes, Model 4. Mediation of independent variable/predictor X on outcome Y by mediator(s) M (Hayes, 2018) used standardized variables to aid interpretability. We controlled for age and body mass index (BMI) by including them as covariates. Each model posited DERS‐16 as X, TRIS as M_1_ and OCI‐R as M_2_. Outcomes (Y_1–5_) were EDS, AUDIT, EDE‐Q, SS, and YFAS symptom count. PROCESS is based on ordinary least squares regression, and produces path coefficients for the total effect (X on Y; *c*), direct effect (X on Y, adjusted for Ms; *c´*), and indirect effect(‐s) (X on Y through Ms; *ab*), the latter using bias‐corrected bootstrap confidence intervals for indirect effects based on 10,000 bootstrap samples, and using 95% confidence intervals (CI). CIs completely over or below zero indicate the presence of an indirect effect. An indirect effect in the absence of a direct effect indicates “full mediation,” while an indirect effect in combination with a significant direct effect indicates “partial mediation.”

## 3 RESULTS

Table 1 shows descriptive statistics and gender comparisons of sample characteristics as well as all psychological and addictive‐like variables. Females scored significantly higher on food addiction, self‐starvation, binge eating, and exercise dependence, with small effect sizes, as well as on emotion dysregulation with a medium effect. Table 2 shows whole‐group intercorrelations among the latter two variable groups. OCI‐R and TRIS correlated significantly with each other and with DERS‐16, and all three correlated significantly with the addictive‐like behaviors, except that impulsivity was not related to exercise dependence. The addictive‐like behaviors were also interrelated, most notably food addiction with binge eating and self‐starvation, as well as binge eating and self‐starvation. Alcohol use was not related to food addiction, and exercise dependence was not significantly associated with binge eating.

**TABLE 2:** Intercorrelations between study variables with medium‐effect (≥.30) associations in bold for emphasis

	DERS‐16	TRIS	OCI‐R	AUDIT	EDS	YFAS	SS
TRIS	**.42** *						
OCI‐R	**.56** *	.28*					
AUDIT	.24*	.26*	.20*				
EDS	.14*	.01	.22*	.14*			
YFAS	**.39** *	.26*	**.32** *	.10	.13*		
SS	**.41** *	.17*	**.41** *	.19*	.28*	**.61** *	
EDE‐Q Binge eating	**.31** *	.23*	.28*	.15*	.05	**.54** *	**.36** *

BMI = body mass index; TRIS = Trait Rash Impulsiveness Scale; OCI‐R = Obsessive‐Compulsive Inventory—Revised; DERS‐16 = Difficulties in Emotion Regulation Scale‐16; YFAS = Yale Food Addiction Scale; SS = Self‐Starvation Scale; AUDIT = Alcohol Use Disorders Identification Test; EDS = Exercise Dependence Scale; EDE‐Q = Eating Disorders Examination Questionnaire.

* <.01.

**FIGURE 1:**
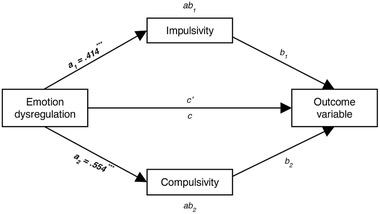
Mediation model showing conceptual design of each mediation analysis, and reporting paths *a*_1_ and *a*_2_, which are the same regardless of outcome

Figure 1 shows the mediation model structure, as well as the *a*_1_ and *a*_2_ paths, showing significant associations between emotion dysregulation and impulsivity and compulsivity. Since there were a few significant gender differences, we ran the mediation models separately also, but found no significant differences between model coefficients (data not shown) and therefore only present full‐group results.

Mediation results for all outcomes are shown in Table 3, where all outcomes were associated with at least one significant indirect path. Exercise dependence was the only outcome to be fully mediated (i.e., *c*´ was not significant), which was through compulsivity. All other outcomes retained significant direct paths, although attenuated by inclusion of the mediators (i.e., partial mediation).

**TABLE 3:** Mediation results using emotion dysregulation (DERS‐16) as independent variable, impulsivity (TRIS) and compulsivity (OCI‐R) as mediator variables, and the five outcome domains: exercise dependence (EDS), alcohol use (AUDIT), binge eating (EDE‐Q), self‐starvation (SS), and food addiction (YFAS)

Outcome	*b*_1_	*b*_2_	*ab*_1_ (CI)	*ab*_2_ (CI)	*c*	*c*’
variable	Impulsivity → outcome	Compulsivity → outcome	via Impulsivity	via Compulsivity		
EDS	–0.075	**0.204** ***	–.031 (–0.079 to 0.011)	**0.113 (0.045–0.185)**	**0.135** **	0.053
AUDIT	**0.185** ***	0.073	**0.077 (0.036–0.125)**	0.041 (–0.033 to 0.130)	**0.241** ***	**0.124** *
EDE‐Q binge eating	**0.114** *	**0.158** **	**0.047 (0.011–0.089)**	**0.088 (0.023–0.159)**	**0.307** ***	**0.172** **
SS	–0.018	**0.256** ***	–0.008 (–0.054 to 0.033)	**0.142 (0.079–0.217)**	**0.417** ***	**0.283** ***
YFAS	**0**.**109** *	**0.149** ***	**0.045 (0.002–0.094)**	**0.083 (0.020–0.159)**	**0.392** ***	**0.264** ***

EDS = Exercise Dependence Scale; AUDIT = Alcohol Use Disorders Identification Test; EDE‐Q = Eating Disorders Examination Questionnaire; SS = Self‐Starvation Scale; YFAS = Yale Food Addiction Scale.

*Note*: Significant direct (also marked with *) and indirect paths (CI not including ±0) are in bold.

* *p *< .05.

** *p* < .01.

*** *p* < .001.

AUDIT was the only outcome to be (partially) mediated via impulsivity alone, and self‐starvation was the only outcome to be (partially) mediated via compulsivity alone; all other outcomes had significant indirect effects via compulsivity. For food addiction and binge eating, both indirect paths had CIs completely above zero and *c*´ was significant; that is, partial mediation was present.

## 4 DISCUSSION

The present study used a community sample to investigate associations between weight‐affecting addictive‐like behaviors (together with alcohol use) and the traits of emotion dysregulation, impulsivity, and compulsivity. The study also tested a mediational model whereby dysregulated emotion was hypothesized to create an urge to escape from or modify emotional discomfort by engaging in addictive‐like behaviors, the choice of which is dependent on levels of impulsivity and compulsivity.

Results suggested that the three eating‐related addictive‐like behaviors food addiction, self‐starvation, and binge eating were strongly correlated with each other. This may be because they tend to reinforce one another (e.g., hunger caused by self‐starvation increases risk for eating more than intended, regardless of whether this involves “grazing” or binge eating), because they share morphological similarities (all involve over‐ or undercontrolled ingestion of food), and because all were associated with emotion dysregulation directly and mediated via compulsivity. The results raise the question of whether these eating‐related addictive‐like behaviors share functional characteristics with regard to their capacity for short‐term emotion regulation if enabled by trait compulsivity.

Importantly, results were consistent with the mediational model that was tested. Emotion dysregulation retained direct associations with all outcome variables except exercise dependence, which was fully mediated through compulsivity. In the case of self‐starvation, food addiction, and binge eating, emotion dysregulation was highly related to each outcome when primarily mediated by compulsivity. In other words, when difficulties with emotion regulation were linked to higher levels of compulsivity, addictive‐like behaviors were scored higher. An interesting finding was that food addiction and binge eating were independently associated with all three constructs, albeit somewhat less with impulsivity than emotion dysregulation and compulsivity. Interestingly, a recent study (Liu et al., 2021) found that trait negative urgency (a facet of impulsivity) was associated with food addiction (as measured using the YFAS) in the presence of lower cognitive flexibility (a component of compulsivity). Although Liu and coworkers did not consider emotion dysregulation, both sets of results suggest that compulsivity and impulsivity may interact to increase risk, which may be an interesting avenue for future research in relation to binge eating. Alcohol dependence was the only outcome variable with a significant indirect effect via impulsivity only, suggesting that poor emotion dysregulation in the presence of impulsivity specifically may increase risk for problematic drinking. In contrast, meta‐review results (Lee et al., 2019) found that while impulsivity was implicated in alcohol addiction, so was compulsivity. However, in that study both constructs were not examined together in such a way as to allow inspection of associations between the trait and the disorder over and above the effect of the other, as was done in the present study.

We also found small‐to‐medium gender differences in mean scores, such that females scored higher on emotion dysregulation, a finding consistent with previous literature and possibly due to different item functioning between genders (Anderson et al., 2016). Also, we found gender differences in the three eating‐related addictive‐like behaviors of food addiction, self‐starvation, and binge eating, as well as slightly higher exercise dependence, which may not be surprising given the higher presence of eating disorder symptomatology in females.

The present study supports and extends previous work suggesting that the interplay of impulsivity, compulsivity, and emotion regulation may be relevant to understand in addictive‐like behaviors. Although we cannot infer causality, our mediation model results suggest that future research may profitably investigate similar ideas longitudinally or experimentally. The interaction of these variables may underlie the propensity of patients to substitute one symptom for another to aid emotion regulation, as has been shown in a clinical eating disorder sample (Garke et al., 2019), as long as the behaviors share features of being sustained and repetitive vs. rash and reactive. As Robbins et al. (2012) have pointed out, impulsivity and compulsivity can interact to shape the expression of behavior; in our data, measures of these traits were significantly correlated, attesting to their partial overlap. In the case of eating disorders, the present findings are consistent with several eating disorder symptoms (binge eating, self‐starvation, compulsive exercise) having addictive qualities, suggesting that there may be important underlying similarities between eating disorders and other addictive‐like behaviors. This could possibly be due to how the impulsive and compulsive nature of eating disorder symptoms can modify dysregulated emotional states and potentially make them more tolerable. However, it is important to note that although our study modeled a plausible direction of effect, reverse influences are certainly possible, such as irregular or disturbed eating patterns affecting emotionality or increasing compulsive tendencies, and such factors could be taken into account in future studies using, for example, experimental designs.

### 4.1 Limitations

The present study has a number of limitations. It was based on a community convenience sample with a higher proportion of females, using a service that is mainly advertised in student populations but where we cannot know how many were actually active students, which limits generalizability. Although the growing transdiagnostic and dimensional view of mental disorders suggests that associations between constructs can profitably be studied in both clinical and nonclinical samples, the present findings need to be replicated and extended to wider populations. Moreover, although we chose measures explicitly operationalizing addictive‐like behaviors based on DSM addiction criteria, our measures are also likely to capture general psychiatric problems, which means that some of the variance is likely not to be addiction‐specific. Additional measures and samples could increase precision in validly measuring the addictive qualities of each behavior. A further limitation is that the OCI‐R is not designed as a trait measure, although it has been shown to correlate significantly with obsessive‐compulsive personality (Wetterneck et al., 2011); explicit measures of trait compulisivity may however yield different results. Also, it may be interesting to investigate DERS subscales to study whether different aspects of emotion dysregulation may be differentially relevant in relation to impulsivity, compulsivity, and addictive‐like behaviors, something we could not do due to power concerns. Even so, given the number of tests performed, Type I errors cannot be ruled out, which suggests results should be treated with caution, and our concurrent design precludes causal interpretations concerning the development of addictive‐like behaviors.

### 4.2 Theoretical and clinical implications

Despite the limitations of the present study, our results raise important questions for future research and clinical practice. As many others have argued (Chamberlain et al., 2018; Markon et al., 2011), it may be important to consider reevaluating established systems of psychiatric classification based on discrete symptoms and instead consider adopting a more dimensional approach. Central constructs in the present study could aid the formation of important dimensions along lines discussed by Chamberlain et al. (2018) and Robbins et al. (2012). It is possible that knowledge about the extent to which weight‐affecting behaviors show addictive qualities and act in the service of emotion regulation may aid treatment planning and outcome prediction. The finding that weight‐affecting behaviors in nonclinical groups have clear addictive qualities may aid understanding why such behaviors can be difficult to change. Clinically, it may be important for therapists who are trying to help eating disorder patients or patients with obesity to focus on confusing and uncomfortable emotional states, since patients may be using impulsive, and especially compulsive behaviors in an addictive‐like manner to help regulate their emotions (Brooks et al., 2017). Exploration, clarification, and differentiation of affective states, as well as interventions that limit impulsivity and especially compulsivity may be important in order to break ingrained patterns of maladaptive behavior.

### PEER REVIEW

The peer review history for this article is available at https://publons.com/publon/10.1002/brb3.2458

## Data Availability

The data that supports the findings of this study is available from the corresponding author upon reasonable request.
